# Molecular Mechanisms of Phthalates in Depression: An Analysis Based on Network Toxicology and Molecular Docking

**DOI:** 10.3390/ijms26178215

**Published:** 2025-08-24

**Authors:** Ruiqiu Zhang, Hairuo Wen, Zhi Lin, Bo Li, Xiaobing Zhou, Qingli Wang

**Affiliations:** 1 National Institutes for Food and Drug Control, Chinese Academy of Medical-Sciences and Peking Union Medical College, Beijing 100730, China; 2 National Center for Safety Evaluation of Drugs, National Institutes for Food and Drug Control, Beijing 100176, China; 3 State Key Laboratory of Drug Regulatory Science, Beijing 102629, China

**Keywords:** phthalates, network toxicology, depression, molecular docking, endocrine resistance

## Abstract

This study investigated the molecular mechanisms by which phthalates induce depression, utilizing network toxicology and molecular docking techniques. By integrating the TargetNet, GeneCards, and PharmMapper databases, 658 potential target genes of phthalates were identified. Additionally, 5433 depression-related targets were retrieved from the GeneCards and OMIM databases. Comparative analysis revealed 360 common targets implicated in both phthalate action and depression. A Protein-Protein Interaction (PPI) network was constructed using the STRING database. Subsequently, the CytoHubba plugin (employing the MCC algorithm) within Cytoscape was used to screen the network, identifying the top 20 hub genes. These core genes include AKT1, CASP3, TNF, TP53, BCL2, and IL6, among others. Validation on the GEO dataset (GSE23848) revealed that the expression of multiple core genes was significantly upregulated in patients with depression (*p* < 0.05). Gene Ontology (GO) and Kyoto Encyclopedia of Genes and Genomes (KEGG) enrichment analyses indicated that phthalates mainly regulate biological processes such as extracellular stimulus response, lipopolysaccharide metabolism, and chemical synaptic transmission. Depression is mediated by the AGE-RAGE signaling pathway (a complication of diabetes), lipids and atherosclerosis, Endocrine resistance, and the PI3K-Akt signaling pathway. Molecular docking confirmed that phthalates have strong binding activity with key targets (CASP3, TNF, TP53, BCL2, IL6). These findings present a novel paradigm for evaluating the health risks posed by environmental pollutants.

## 1. Introduction

Phthalates are the most commonly used plasticizers, accounting for approximately 80% of the total plasticizers and are widely used in the production of plastic products [1]. It is widely added to polyvinyl chloride (PVC) flooring, wires and cables, wallpaper, food packaging materials, medical devices, children’s toys, as well as personal care products such as perfumes, nail polish and shampoos [2]. Common commercial homologues include di (2-ethylhexyl) phthalate (DEHP), dibutyl phthalate (DBP), diethyl phthalate (DEP), butyl benzyl phthalate (BBP), and diisononyl phthalate (DINP) [3], etc. Among them, DEHP and its metabolite mono (2-ethylhexyl) phthalate (MEHP) have the highest detection frequency and concentration in the human body and have been used as the most commonly used exposure biomarkers in biological samples such as urine, serum and breast milk. Phthalates are small-molecule organic compounds with extremely unstable physical and chemical properties [4], they cannot bond with plastic polymers through chemical bonds. Under certain physical and chemical conditions (such as temperature, pH value, etc.), they can easily migrate from plastic products to the environment, causing exposure to the environment [5,6,7]. Over the past 50 years, although the transfer of phthalates from packaging materials or containers to food and beverages [8], which leads to human exposure, has drawn attention, the usage of phthalates in daily necessities and food packaging materials remains extremely large [9]. Plastic, as one of the most commonly used food packaging materials, is widely applied in the production of food containers, fast food boxes, baby bottles, water cups, etc. [10,11]. A large number of studies have shown that dietary behavior is the main way for the general population to be exposed to phthalates [12,13], especially the consumption of fast food and convenience food [14]. A few studies have suggested that humans may be exposed to phthalates through phthalate-polluted air [15]. Moreover, studies have found that floor adhesives can release diphenyl phthalates into the indoor air [16]. Phthalates enter human tissues through ingestion, inhalation, and dermal absorption. Their metabolites, such as mono—(2-ethylhexyl) phthalate (MEHP), are frequently detected in urine, serum, and breast milk [17,18]. As confirmed endocrine disruptors (EDCs), phthalates can interfere with hormone signaling pathways, especially those involving estrogen and thyroid receptors [6,19,20]. Existing research mainly focuses on reproductive toxicity and developmental disorders [21,22], the evidence linking phthalates to neuropsychiatric disorders, especially depression, remains fragmented and understudied in terms of mechanism.

Depression is a debilitating mental disorder affecting over 300 million people worldwide [23,24], characterized by dysregulation of neurotransmitter systems (such as serotonin and dopamine) and neuroinflammation [25]. Epidemiological studies indicate that individuals with depressive symptoms exhibit elevated levels of phthalate metabolites [26,27], although the causal mechanism remains unclear. Notably, women exhibit higher body burdens of phthalates and greater susceptibility to depression [28,29], suggesting a potential endocrine-mediated pathway. The bio-accumulative nature of chronic low-dose phthalate exposure, coupled with its increasing use in industrialized societies, underscores the urgent need to elucidate its neurotoxic potential. Current limitations in phthalate risk assessment include methodological gaps where previous analyses have heavily relied on epidemiological correlations or single-target molecular studies, lacking mechanistic integration at the systems level; pathway ambiguity in key depression-linking processes—such as neuroendocrine disruption, oxidative stress, and synaptic dysfunction—which remain poorly defined; and uncharacterized interactive effects of EDCs mixtures, where combined human exposure may amplify neurotoxicity through undocumented cross-talk mechanisms.

To address these limitations, this study employs a network toxicology and molecular docking approach—a paradigm successfully applied in assessing triclosan-induced osteotoxicity [30]. We integrate multi-database target mining, protein-protein interaction network analysis, and computational validation to identify shared targets between phthalates and depression pathogenesis. Core signaling pathways are deciphered through functional enrichment analysis, while binding affinities of phthalates to hub targets are computationally validated. This methodology establishes a novel framework for rapid risk prioritization of environmental neurotoxicants. Additionally, this study offers insights into efficient strategies for assessing the toxicity of environmental pollutants and provides guidance for regulatory policies and the informed use of antimicrobial agents.

## 2. Results 

### 2.1. Target Retrieval

**Figure 1:**
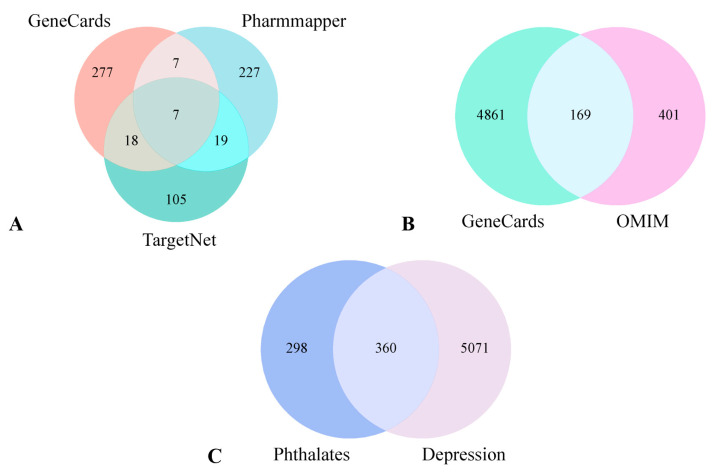
Target identification workflow. Target collection for phthalates (**A**). Target collection for depression (**B**). Venn diagram showing intersecting targets between phthalates and depression (**C**).

A total of 658 phthalates target genes were identified from three databases: TargetNet, GeneCards, and Pharmmapper, as shown in Figure 1A. A total of 5030 and 570 depression—related targets were retrieved from GeneCards and OMIM, respectively. After removing duplicates, 5431 unique depression targets were obtained, as shown in Figure 1B. The intersection of disease genes with triclosan targets resulted in 360 shared genes, as shown in Figure 1C. These 360 targets are strongly associated with phthalates—induced depression.

### 2.2. PPI Network of Shared Genes and Discovery of Core Targets

**Figure 2:**
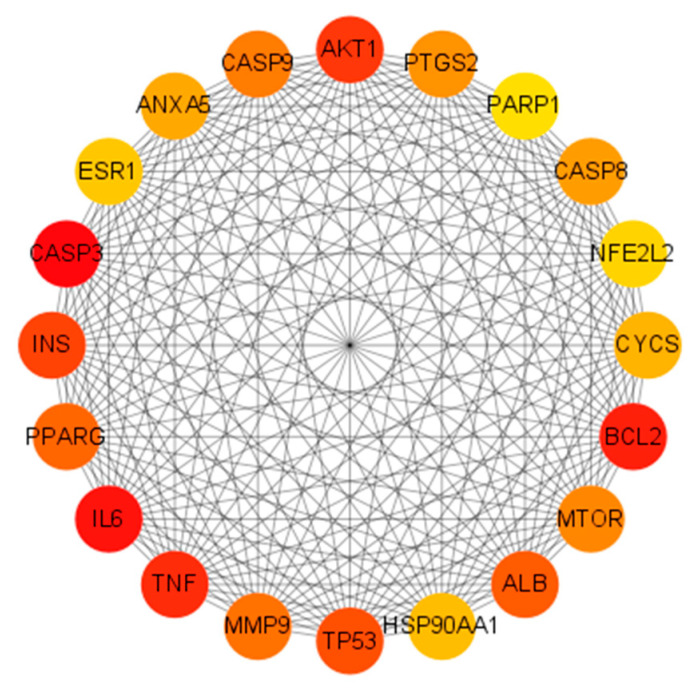
Core genes identified using the MCC algorithm. The network diagram represents complex interactions among 20 core genes, highlighting their functional connections. Node color intensity (red → yellow) indicates MCC score (red = highest).

A PPI network of the 360 shared targets was constructed using the STRING database, resulting in 353 nodes, 7125 edges, and an average node degree of 40. The TSV files were exported from STRING and imported into Cytoscape software 3.10.3 for further analysis. Key genes were screened using the CytoHubba plugin in Cytoscape. The Matthews correlation coefficient (MCC) algorithm, recognized as a robust scoring method, was applied in CytoHubba to identify and visualize the top 20 core genes: AKT1, PTGS2, PARP1, CASP8, NFE2L2, CYCS, BCL2, MTOR, ALB, HSP90AA1, TP53, MMP9, TNF, IL6, PPARG, INS, CASP3, ESR1, ANXA5 and CASP9, as shown in Figure 2.

### 2.3. Randomized Dataset Validation of Core Genes

**Figure 3:**
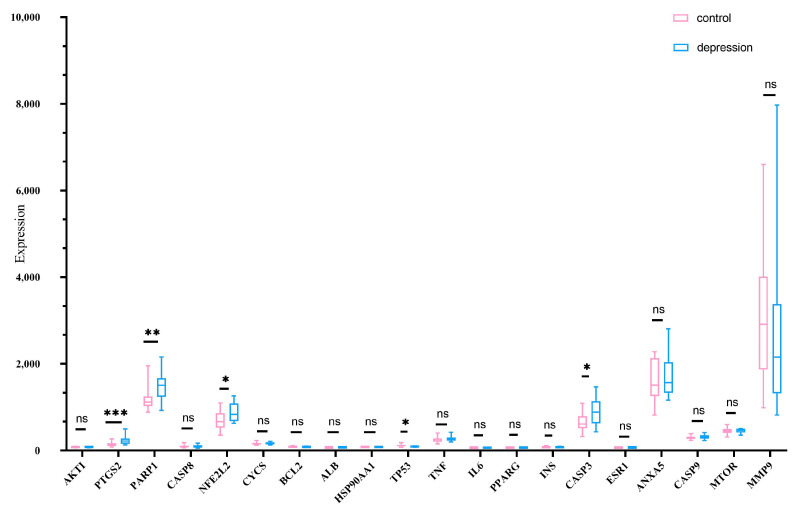
Differential expression profiles of 20 core genes across data sets from individuals with depression and from controls without depression. Blue: depression; Pink: control. * *p* < 0.05; ** *p* < 0.01; *** *p* < 0.001; ns: There was no statistical significance.

To validate the biological relevance of the 20 core genes identified from the PPI network, we analyzed their expression profiles in the GSE23848 dataset, which includes peripheral blood samples from patients with major depressive disorder (MDD). This step bridges the in-silico prediction with clinical transcriptomic evidence. The GSE23848 dataset was randomly selected to validate the expression profiles of core genes. This dataset includes 16 samples from healthy people as the control group and 20 samples from patients with depression as the disease group. Differences in core gene expression between the two groups were analyzed using the t- test. Results showed significant differences (*p* < 0.05) in the expression levels of several genes, with higher expression in the disease group compared with the controls, as shown in Figure 3.

### 2.4. Enrichment Analyses for GO Terms and KEGG Pathways

**Figure 4:**
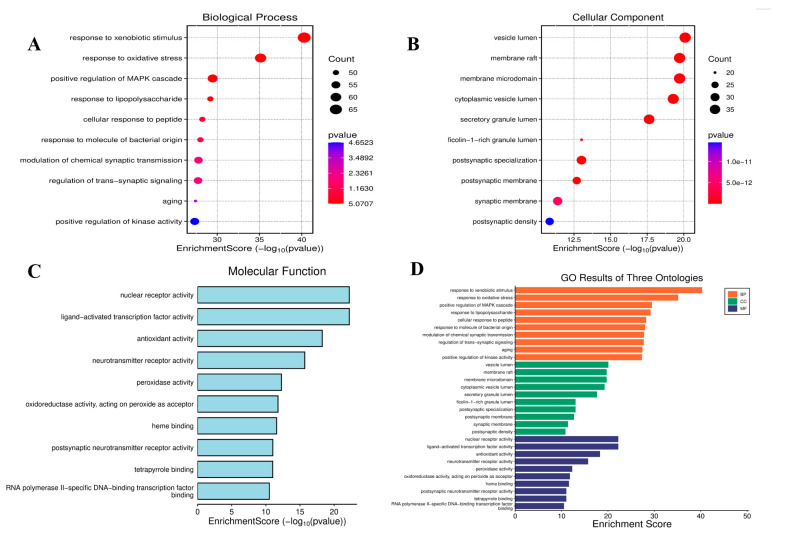
GO enrichment analysis of 360 shared targets. (**A**) Top 10 biological processes (BP), (**B**) cellular components (CC), (**C**) molecular functions (MF) and (**D**) Bar graph of GO analysis. Bar colors represent −log10 (*p*-value).

To elucidate the biological mechanisms by which phthalates exacerbate depression, GO term and KEGG pathway enrichment analyses were performed on 360 common targets using the “clusterProfiler” package https://www.bioinformatics.com.cn/?p=1 (accessed on 21 August 2025) in R. According to the adjusted ascending *p* value, the top ten GO-BP terms are related to processes such as the cell’s response to exogenous stimuli, lipopolysaccharide metabolism, chemical synaptic transmission, and the regulation of transsynaptic signal transduction, as shown in Figure 4A. The top ten GO-CC terms involve cellular components such as extracellular matrix containing collagen, membrane rafts, membrane microregions, secretory granule cavities, vesicle cavities, and synaptic membranes, as shown in Figure 4B. Similarly, the top ten GO-MF terms are related to molecular functions, including receptor-ligand activity, cytokine receptor binding, cytokine activity and neurotransmitter activity, as shown in Figure 4C.

**Figure 5:**
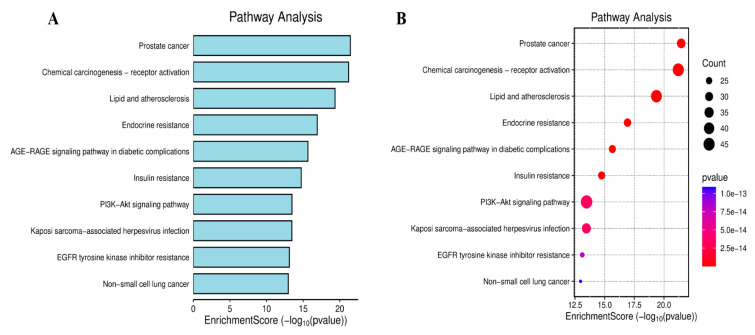
Analysis of KEGG pathways. Bar chart of KEGG analysis (**A**). Bubble chart of KEGG analysis (**B**). KEGG pathway enrichment analysis revealed that it mainly involved diabetic complications, lipids and atherosclerosis, endocrine resistance, steroid hormone biosynthesis, etc.

KEGG pathway enrichment analysis identified 199 pathways (adjusted *p* < 0.05), mainly involving AGE-RAGE signaling in diabetic complications, lipids and atherosclerosis, endocrine resistance, steroid hormone biosynthesis, PI3K-Akt and various cancer-related pathways. As shown in Figure 5A,B, the top ten use bar charts and bubble charts to visualize the KEGG pathway.

### 2.5. Molecular Docking of Phthalates with Depression Core Targets

**Figure 6:**
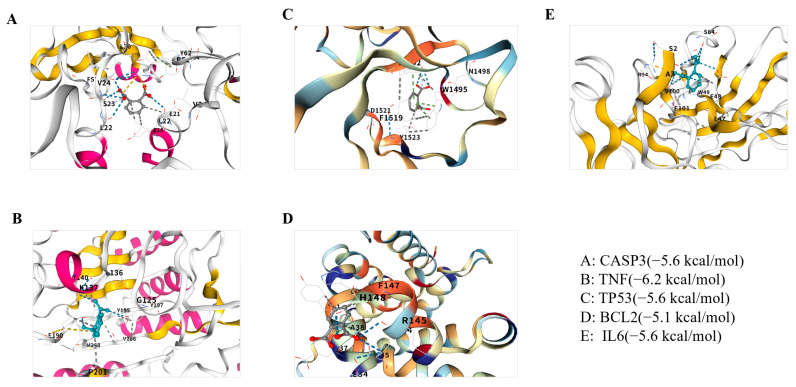
Molecular docking results of b phthalates with CASP3, TNF, TP53, BCL2 and IL6, displaying the lowest binding energies in 3D formats. (**A**): CASP3; (**B**): TNF; (**C**): TP53; (**D**): BCL2; (**E**): IL6

Molecular docking analyses were conducted to investigate the interactions between phthalates and five core targets: CASP3 (PDBID: 6X8I), TNF (PDBID: 7YPC), TP53 (PDBID: 6VA5), BCL2 (PDBID: 6FBX), and IL6 (PDBID: 79HS). Phthalate is a family of benzene derivatives that are formed by esterification of phthalic acid. Phthalates are added to increase the flexibility and softness of commercial plastics, which are incorporated into a wide variety of consumer goods. Ingested phthalates may exhibit estrogenic or antiandrogenic effects or they may act as endocrine disruptors. Exposure to phthalate may increase the risk of certain cancers. So, in this manuscript, we have chosen it to explore its connection with depression. The docking results of CB-Dock2 showed that the binding energies between phthalates and target proteins were all less than −5 kcal/mol, suggesting strong binding activity. The binding energies of phthalates to five key target molecules, namely CASP3, TNF, TP53, BCL2 and IL6, are −5.6 kcal/mol, −6.2 kcal/mol, −5.6 kcal/mol, −5.1 kcal/mol and −5.6 kcal/mol respectively. Generally, a binding energy < 0 indicates binding activity, whereas values below −5.0 kcal/mol indicate strong binding activity, with lower values denoting stronger interactions. The results demonstrated strong affinities between phthalates and the five core targets, indicating spontaneous binding and suggesting their critical role in the molecular mechanisms of phthalates induced depression. The docking results were visualized in 3D using CB-Dock2, as shown in Figure 6.

## 3. Discussion

This study employed a systematic and standardized bioinformatics approach to analyze multiple databases, integrating gene target and compound data to identify 360 potential targets related to phthalates-induced depression. A PPI network was constructed using STRING and Cytoscape data, identifying the top five core targets through the MCC algorithm: CASP3, TNF, TP53, BCL2, and IL6. These targets are significantly enriched in neuroinflammation, apoptosis and synaptic function regulatory pathways, suggesting that phthalates may induce depression by disrupting the homeostasis of the “neuroendocrine-immune” network. It is worth noting that this study has methodological consistency with Wang et al. [30] research on triclosan-induced osteotoxicity. Both found that the PI3K-Akt pathway and endocrine resistance are the core hubs of EDCs pathogenesis, suggesting that different environmental pollutants may interfere with physiological functions through conserved signaling axes.

CASP3, as an executor of apoptosis, is abnormally activated in the prefrontal cortex of patients with depression [31]. In this study, the GSE23848 dataset showed that its expression was significantly upregulated (*p* < 0.01), and the molecular docking binding energy reached −5.6 kcal/mol, indicating that phthalates may directly activate CASP3 and trigger neuronal apoptosis [32]. This echoes the discovery of CASP3 as a TOP20 core target in the triclosan osteotoxicity study, indicating that EDCs may share the apoptotic regulatory mechanism [30]. TNF is a key mediator of neuroinflammation. This study confirmed that it has the strongest binding energy with phthalates (-6.2 kcal/mol). Overexpression of TNF can disrupt the integrity of the blood-brain barrier and promote the infiltration of inflammatory factors into the central nervous system, which is directly associated with the KeGG-enriched “lipids and atherosclerosis” pathway, suggesting that EDCs may trigger chronic inflammation in different tissues through a similar mechanism [33,34]. TP53 exhibits non-classical functions in depression. Besides regulating DNA repair, it also participates in the activation of tryptophan metabolic enzymes, resulting in a reduction in 5-HT synthesis [35,36]. The expression of TP53 was upregulated (*p* < 0.05) and strongly bound to phthalates (−5.6 kcal/mol), which might be a key node connecting neurotransmitter imbalance and cellular stress. The abnormalities of BCL2/IL6 respectively point to mitochondrial apoptotic pathways [37,38] and neuroimmune dysregulation. Down-regulation of BCL2 will amplify oxidative stress damage [39], while excessive secretion of IL6 can activate microglia [40], forming a vicious cycle of “inflammation-oxidative stress”.

GO analysis indicated that phthalates mainly intervened in the cell’s response to exogenous stimuli and chemical synaptic transmission. This is highly consistent with epidemiological studies. Phthalates can pass through the blood-brain barrier and accumulate in emotional regulation regions such as the amygdala and hippocampus, interfering with glutamatergic/GABAergic synaptic plasticity [41]. It is worth noting that the significant enrichment of lipopolysaccharide metabolism suggests that intestinal flora imbalance may be a potential mechanism. Phthalates have been proven to disrupt the intestinal barrier, promoting lipopolysaccharide (LPS) to enter the bloodstream and triggering systemic inflammation [42]. KEGG pathway analysis further reveals multiple pathogenic networks. In diabetic complications, advanced glycation end products activate NF-κB by binding to the RAGE receptor in the AGE-RAGE signaling pathway [43], driving neuroinflammation. Besides, phthalates, as classic EDCs, can interfere with the negative feedback regulation of the hypothalamic-pituitary-adrenal (HPA) axis by competitively binding to estrogen receptors, thyroid hormone receptors [44], etc. The above research results all indicate the influence of phthalates in depression and the results of molecular docking also confirmed this point.

Our study has several limitations. The primary limitation is the lack of in vivo experiments to evaluate the effect of phthalates on depression, which would validate the core targets and effector pathways, facilitating the development of preventive and therapeutic strategies. Additionally, phthalates frequently coexist with environmental pollutants such as bisphenol A (BPA) and flame retardants in real-world settings. Future studies should therefore employ systems toxicology approaches to evaluate the synergistic neurotoxicity of such combined exposures. The current network model does not encompass phthalate metabolites (e.g., MEHP), which may represent a critical factor underlying nonlinear dose-response relationships. Finally, we suggest that everyone maintain a healthy lifestyle and minimize exposure to phthalates, as this may harm one’s health.

## 4. Methods and Materials

### 4.1. Collection of Phthalates Targets

We searched PubChem for “phthalates” to obtain its chemical structure and smile format. Phthalate and its structural formulas are shown in Table 1. Therefore, we retrieved potential phthalates targets from the GeneCards, Pharmmapper and TargetNet databases. Then use string and universal protein resource (UniProt) to standardize the target name. We use UniProt’s ID mapping to standardize the identifiers from GeneCards, Pharmmapper and TargetNet in order to convert all entries into standardized genetic symbols. Redundant targets are removed through automatic cross-reference, and interactions are filtered by retaining only high-confidence entries (STRING confidence score > 0.7). The merged and filtered targets were used to construct the phthalates target library.

### 4.2. Depression Target Retrieval

Using “depression” as the key word, depression related targets were retrieved from GeneCards and OMIM. Then the targets are merged into a unified depression target data set. The screening criteria included: the correlation score of GeneCards was ≥1.

### 4.3. ”Phthalates-Depression” Network Plot

The targets of phthalates and depression are visualized in the Venn Diagram, and their cross-targets are identified using the “Venn Diagram” package https://www.bioinformatics.com.cn/plot_basic_proportional_2_or_3_venn_diagram_028 (accessed on 21 August 2025) in R. Detailed information on each gene set in the Venn Diagram can be found in Supplementary Table S1.

### 4.4. Construction of Protein-Protein Interaction Networks

Shared genes between phthalates and depression targets were imported into the STRING database to retrieve protein-protein interactions (PPIs) and construct a PPI network under the following parameters: species set to Homo sapiens and interaction score > 0.7 (high confidence). STRING TSV files were imported into Cytoscape, where network topology analysis was conducted using the CytoHubba plugin to identify core proteins.

### 4.5. External Gene Expression Dataset Validation

Gene expression matrices were retrieved from the Gene Expression Omnibus (GEO) database with the keywords “depression” and “Homo sapiens”. The average age of the depression group was 41.8 ± 12.4 years, while that of the control group was 39.9 ± 10.7 years. There was no statistically significant difference (*p* = 0.52). Differential expression of core targets between normal and diseased samples was analyzed using t-test and visualized as box plots.

### 4.6. Gene Ontology (GO) and Kyoto Encyclopedia of Genes and Genomes (KEGG) Enrichment Analyses

To elucidate the mechanism by which phthalates induced depression, intersecting target gene abbreviations for phthalates and depression were converted into Entrez IDs using the “org.Hs.eg.db” package https://www.uniprot.org/ in R. These IDs were subsequently subjected to GO functional enrichment and KEGG pathway analysis using the “clusterProfiler”, “enrichplot“ and “ggplot2” packages https://www.bioinformatics.com.cn/basic_local_go_pathway_enrichment_analysis_122 (accessed on 21 August 2025) in R. Enrichment results with both *p*-values and adjusted *p*-values (q-values) below 0.05 were selected, yielding the top ten GO processes and top ten KEGG pathways ranked by descending q-value.

### 4.7. Molecular Docking of Phthalates to Core Targets

Molecular docking was used to analyze the intermolecular interactions between phthalates and the core targets identified in this study by predicting binding modes and affinities. Core targets were designated as receptors, whereas phthalates served as the ligand for docking validation. The isomeric SMILES of phthalates was retrieved from the PubChem database, and the Protein Data Bank (PDB) file of the core target protein was down-loaded using its UniProt ID from the PDB database. CB-Dock2 was used to preprocess the small-molecule ligand and target protein, including energy optimization, hydrogen addition, water molecule removal, and energy minimization. The docking site with the lowest Vina score was selected as the optimal binding mode. Binding energies were calculated, and the binding mode was visualized in 3D.

### 4.8. Data Sources

All databases were accessed between March and August 2025. The specific version is shown in Table 2.

## 5. Conclusions

This study integrates network toxicology and molecular docking to elucidate phthalate-induced depression mechanisms, revealing four key findings: (1) Multi-database screening identified 360 shared targets from 658 phthalate targets and 5433 depression targets; (2) PPI network analysis prioritized CASP3, TNF, TP53, BCL2, and IL6 as core hub genes, with GEO validation (GSE23848) confirming their significant dysregulation in patients; (3) Functional enrichment demonstrated that phthalates mediate neuroinflammation and synaptic dysfunction by regulating extracellular stimulus response, LPS metabolism, and synaptic transmission through AGE-RAGE, lipid/atherosclerosis, endocrine resistance, and PI3K-Akt pathways; (4) Molecular docking confirmed strong binding affinity (<−5 kcal/mol) between phthalates and core targets, indicating spontaneous functional interference. Collectively, we establish the first “phthalate-depression” interaction model, highlighting neuroendocrine-immune disruption and inflammatory dysregulation as central mechanisms. This integrated strategy provides a pioneering framework for environmental neurotoxicity assessment, calling for future studies on combined EDC exposures and in vivo validation of core targets.

## Figures and Tables

**Table 1:** The chemical formula and structural formula of phthalate.

Smile	Structure
C1=CC=C(C(=C1)C(=O)[O-])C(=O)[O-]	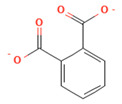

**Table 2:** Data source and access link.

Database Name	Access Address
TargetNet	Index-Home-TargetNet
PharmMapper	https://www.lilab-ecust.cn/pharmmapper/
GeneCards	https://www.genecards.org/
OMIM	https://www.omim.org/
UniProt	https://www.uniprot.org/
STRING	https://cn.string-db.org/
GEO	https://www.ncbi.nlm.nih.gov/gds
CB-DOCK2	CB-Dock2: An accurate protein-ligand blind docking tool

## Data Availability

The original contributions presented in this study are included in the article/Supplementary Material. Further inquiries can be directed to the corresponding author.

## Supplementary Materials

The following supporting information can be downloaded at: https://www.mdpi.com/article/10.3390/ijms26178215/s1.
